# Food Insecurity, Local Food Environments, and Incident Dementia Among Older Adults

**DOI:** 10.1093/geroni/igaf122.1689

**Published:** 2025-12-31

**Authors:** Boeun Kim, Sarah Szanton, Suyoung Kwon, Wen Liu, Laura Samuel, Roland Thorpe, Paris Adkins-Jackson

**Affiliations:** University of Iowa, Iowa City, Iowa, United States; Johns Hopkins University, Baltimore, Maryland, United States; George Mason University, Fairfax, Virginia, United States; Penn State University, University Park, Pennsylvania, United States; Johns Hopkins University, Baltimore, Maryland, United States; Johns Hopkins University, Baltimore, Maryland, United States; Mailman School of Public Health, Columbia University, New York, New York, United States

## Abstract

It is well known that food insecurity contributes to disparities in dementia risk; however, no studies have examined the role of food environments in exacerbating the relationship between food insecurity and dementia risk in older adults. We conducted a multi-methods study to examine the associations of food insecurity and food environments with dementia risk and explore how food environments affect food purchasing and healthy eating. We analyzed data from 6,551 participants aged 50+ without dementia in 2014 from the Health Care and Nutrition Study. We conducted individual interviews with 16 community-dwelling adults aged 50+. Food insecurity was assessed in 2013 using the six-item Short Form US Household Food Security Survey Module. Food environmental factors included the county-level price of low-fat milk, as well as low-income, low-food access, and low-vehicle access at Census-tract-level. Incident dementia from 2016 to 2020 was identified using a validated algorithm. Discrete-time proportional hazard models were fitted, adjusting for age, gender, education, racialized group, survey mode, living arrangement, and urbanicity. Residing in neighborhoods characterized by low-income, low-vehicle access, and higher low-fat milk prices was associated with a greater risk of incident dementia. Our qualitative findings further demonstrated food environmental factors relevant to healthy food purchasing and eating—both protective against dementia—including convenience, portion variety, prices, quality, safety, supply, mobility aids, proximity, and transportation. However, food environment factors did not modify the association between food insecurity and dementia risk. These findings underscore the significant role of both food environments apart from food insecurity in shaping dementia risk.

